# The Effect of Timing and Frequency of Push Notifications on Usage of a Smartphone-Based Stress Management Intervention: An Exploratory Trial

**DOI:** 10.1371/journal.pone.0169162

**Published:** 2017-01-03

**Authors:** Leanne G. Morrison, Charlie Hargood, Veljko Pejovic, Adam W. A. Geraghty, Scott Lloyd, Natalie Goodman, Danius T. Michaelides, Anna Weston, Mirco Musolesi, Mark J. Weal, Lucy Yardley

**Affiliations:** 1 Psychology, Faculty of Social, Human, and Mathematical Sciences, University of Southampton, Southampton, Hampshire, United Kingdom; 2 Primary Care and Population Sciences, Faculty of Medicine, University of Southampton, Southampton, Hampshire, United Kingdom; 3 Department of Creative Technology, Faculty of Science and Technology, Bournemouth University, Poole, Dorset, United Kingdom; 4 Faculty of Computer and Information Science, University of Ljubljana, Ljubljana, Slovenia; 5 Redcar & Cleveland Borough Council, Redcar, Yorkshire, United Kingdom; 6 Health and Social Care Institute, School of Health and Social Care, Teesside University, Middlesbrough, Tees Valley, United Kingdom; 7 Fuse, Centre for Translational Research in Public Health, Newcastle University, Newcastle upon Tyne, Tyne and Wear, United Kingdom; 8 Centre for Public Policy and Health, School of Medicine, Pharmacy and Health, Durham University, Stockton on Tees, United Kingdom; 9 Gateshead Council, Gateshead, Tyne and Wear, United Kingdom; 10 Electronics and Computer Science, University of Southampton, Southampton, Hampshire, United Kingdom; 11 Department of Geography, University College London, London, United Kingdom; University of Rochester, UNITED STATES

## Abstract

Push notifications offer a promising strategy for enhancing engagement with smartphone-based health interventions. Intelligent sensor-driven machine learning models may improve the timeliness of notifications by adapting delivery to a user’s current context (e.g. location). This exploratory mixed-methods study examined the potential impact of timing and frequency on notification response and usage of Healthy Mind, a smartphone-based stress management intervention. 77 participants were randomised to use one of three versions of Healthy Mind that provided: intelligent notifications; daily notifications within pre-defined time frames; or occasional notifications within pre-defined time frames. Notification response and Healthy Mind usage were automatically recorded. Telephone interviews explored participants’ experiences of using Healthy Mind. Participants in the intelligent and daily conditions viewed (d = .47, .44 respectively) and actioned (d = .50, .43 respectively) more notifications compared to the occasional group. Notification group had no meaningful effects on percentage of notifications viewed or usage of Healthy Mind. No meaningful differences were indicated between the intelligent and non-intelligent groups. Our findings suggest that frequent notifications may encourage greater exposure to intervention content without deterring engagement, but adaptive tailoring of notification timing does not always enhance their use. Hypotheses generated from this study require testing in future work.

**Trial registration number**: ISRCTN67177737

## Introduction

The potential for digital interventions to effect positive behaviour change has been demonstrated in a number of health domains [1]. Yet, intervention usage is often below desired levels [2,3]. Intervention prompts (e.g. emails, SMS, push notifications) have shown promise for motivating initial enrolment to health behaviour change interventions [4] and evoking repeated intervention use [5–7], particularly when prompts contain feedback, theoretically-informed content or behaviour change techniques [6,8]. Following Fogg’s behavioural model [9], prompts may provide the necessary trigger to engage with intervention content whereas theoretically informed prompt content may provide the necessary motivation to do so.

Smartphones enable on-the-go delivery of intervention content via push notifications that can be delivered at convenient times for the user or when specific intervention content is needed [10,11]. Notifications can also prompt access to more intensive support provided by other platforms [12,13]. However, evidence suggests that users can receive in excess of 50 notifications per day from a variety of apps [14]. Research has also indicated that sending additional push messages in different formats (e.g. email and SMS) may have adverse effects on desired behaviour compared to the use of just one message type [15]. To increase the likelihood that users will attend to intervention notifications it is vital to first identify the factors that enhance or undermine notification response.

Qualitative research suggests that apps may be quickly discarded if notifications are perceived to be irritating or intrusive [16]. Notifications appear to be most acceptable when users are provided with control over if, when, and how they are received, and when notifications are delivered at convenient times that do not disrupt daily routine [16–19]. Current research does not yet provide precise indications about when these convenient times might be or the threshold for when notifications become irritating and intrusive. To optimise the potential impact of notifications from any app it is vital to establish: a) when users are most likely to attend and respond to notifications; b) how many notifications are optimal for increasing engagement.

SMS messages sent at user designated ‘good’ times (versus other random times) were found to have little impact on receptivity to and perceived timeliness of messages [20]. Instead, receptivity and timeliness of SMS messages was influenced by perceptions of the notification content (e.g. interest). It is not clear how well these findings translate to perceptions of smartphone notifications or a health behaviour change context where interest in and motivation to attend to notification content may differ. Tailoring notification delivery to user-designated ‘good’ times also places unnecessary burden on the user. Evidence suggests that users are not able to successfully anticipate timeframes within which they will be available and receptive to receiving notifications and that convenient moments are not necessarily consistent day to day [13,20].

Intelligent, sensor-driven machine learning algorithms enable the timing and content of notifications to fit with and adapt to the users’ current context (e.g. location, physical activity, social interaction, sleep patterns etc.) or health state (e.g. stress, mood, physiological functioning) [21]. To ensure the content of sensor-driven notifications is engaged with, a fundamental question is whether sensor data can determine when users are able and willing to respond to a notification [22–26]. If so, engagement with the content of sensor-driven notifications may be enhanced. While models developed to date show promise, early research to support their accuracy is necessarily conducted in highly controlled, contrived settings where participants are often incentivised to provide reports on the timeliness of notifications that are received several times a day. Anticipation of interruptible moments in some models has also relied on the use of wearable sensors [e.g. 26]. It is not clear how successful these models will be in a naturalistic context where users may be less inclined to respond to notifications and where it is less feasible to harness wearable sensors.

To our knowledge, no study has yet examined the impact of sensor driven notifications informed solely from phone-based sensors and delivered in a real-world public health context. This exploratory study compares the impact of intelligent, sensor-driven notifications with non-intelligent notifications sent within pre-determined timeframes. All notifications were provided by “Healthy Mind”, an Android app-based stress management intervention disseminated in a UK-based public health setting. The aims of the study were to investigate the potential impact of notification timing (intelligent versus non-intelligent notifications) and frequency (daily versus occasional notifications) using a mixed methods approach. Usage patterns provided indications about the strength of any association between notification delivery and notification response or intervention usage. Qualitative data on participants’ experiences of using the app generated potential explanations for when and why notifications were (not) responded to. The Healthy Mind intervention has been described in accordance with the TIDieR checklist [27]. The qualitative components of the study have been reported in accordance with the COREQ criteria for interviews and focus groups [28].

## Method

### Design

Participants were randomised post-baseline to one of three versions of Healthy Mind: intelligent, daily, or occasional. Intelligent notifications were triggered at times when the algorithm predicted that a user was most likely to notice and respond. Opportune times for each user were identified by sampling data from three phone-based sensors: location (GPS), movement (accelerometer), and time of day (clock). The first two notifications were triggered at random, but within designated time and frequency parameters. The timing and frequency of notification triggering was then refined after every notification, that is, the app learned when and in what contexts notifications were responded to most often. Specifically users could receive up to 3 notifications per day between 08.00 and 22.00 hours. Users could customise the time range within which notifications were received. Following this learning period, a model of interruptibility was then built for each user using a Naïve Bayesian classifier that established a relationship between specific contexts and likelihood of notification response. Once the model was trained, the user’s context was sampled every 20 minutes to anticipate the likelihood of notification response.

The classifier utilised location (GPS), movement (accelerometer) and time variables that were derived from the raw sensor readings. Initially, users’ sampled GPS co-ordinates were clustered and averaged within particular time-frames to infer “home” (01.00 to 06.00 hours), “work” (10.00–16.00 hours), and “other” locations. GPS co-ordinates within a 500m radius of “home” or “work” co-ordinates were then labelled as “home” or “work”. Co-ordinates outside of this radius were labelled “other”. Accelerometer X, Y, and Z values were collected for 60s within each 20 minute sampling window. In line with previous activity recognition research, the mean intensity of acceleration, the variance of acceleration and the mean crossing rate were then calculated from the raw accelerometer values to provide a proxy measure of movement [29]. Time variables were hour of day and weekend versus weekday.

The classifier labelled the likelihood of notification response as either *yes* or *no* based on the combined values of the sensed variables. A notification was only triggered if a yes label was returned (i.e. notification response was deemed likely). The relative weighting of each sensed variable within the classifier varied between users. That is, the model of interruptibility was personalised to each individual user. A Naïve Bayesian classifier assumed that variables within each personalised model were unrelated. This means that the relative weighting of each sensed variable did not vary with respect to other variables in the model. For example, the importance placed on a user’s motion within the classifier remained the same regardless of whether the user was designated to be at home, work or other location. The sensing, data processing, and generation of interruptibility models were handled by independent open-source Android libraries [24,30].

Daily and occasional notifications were triggered randomly within a time range of 17.00 to 20.00 hours. As with notifications, users were able to customise this time range. If the time frame specified by the participant did not include 17.00 to 20.00 hours then notifications were triggered at another random time within the limits specified by the participant. The daily version triggered one notification within a 24 hour period. The occasional version triggered one notification within a 72 hour period. The time frame of 17.00 to 20.00 hours was chosen since prior research has indicated that intervention or notification engagement typically occurs during non-working hours [13,17].

### Procedure

Employers were recruited to the study via local UK public health teams, many of whom were involved in workplace health activities via the North East Better Health at Work award (described by [31]). Posters, newsletters and email circulars were used to promote the study to employees, who downloaded Healthy Mind via the Google Play Store. Standard Google guidelines were followed to inform participants about what data was being collected. Data collection took place between September 2014 and February 2015 and the entire study was approved by the University of Southampton ethics committee and research governance office (approval number: 12156). Study procedures were fully automated using LifeGuide and Life Guide Toolbox software (http://www.lifeguideonline.org). Therefore participants provided informed consent to participate in the study electronically. After downloading the app, participants were presented with a participant information screen that provided information about the study. Participants were informed that they could delete the app at any time. Informed consent was provided by clicking ‘next’ on this screen and continuing to complete the baseline self-report measures. This consent procedure was approved by the University of Southampton Ethics Committee and Research Governance Office. Participants were free to use Healthy Mind as often or as little as they wished.

A link to an online feedback survey was sent via email two weeks after initial app download, which included an invitation to participate in a semi-structured telephone interview. Participants were sent a further three email reminders to complete the online feedback survey. All participants who provided consent to be interviewed were contacted via email and/or telephone by LM to arrange the interview. The online feedback survey did not ask participants to explain their reasons for declining to give consent to be interviewed. First contact between participants and interviewers was an email to arrange a convenient date/time to conduct the telephone interview. Thus, no prior relationship between participants and interviewers was established. All interviews were conducted by a female researcher with prior training and experience in conducting qualitative interviews (LM). Interviews lasted between 10 and 32 minutes. Member checks were employed during the interview (e.g. interviewer restated or summarised participants’ accounts to check understanding and prompt further elaboration). No member checks were completed after data analysis.

### Intervention

**Fig 1:**
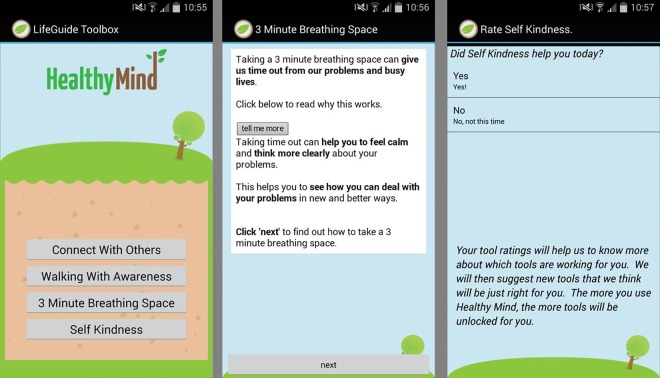
Screen shot of Healthy Mind tool menu screen, tool description screen, and tool rating screen (left to right).

Healthy Mind is a stand-alone Android smartphone application that offers evidence-based tools for managing stress and other negative emotions (see Fig 1). Healthy Mind was created using the Life Guide Toolbox software [32]. The tools provided by Healthy Mind were drawn from mindfulness-based approaches and Cognitive Behavioural Therapy (CBT) (e.g. breathing and meditation practices, monitoring and planning positive experiences, and self-compassion, see S1 Table for a full list and description of the Healthy Mind tools). The content for Healthy Mind was adapted from a pre-existing web-based intervention (Healthy Paths) following a person-based approach [33]. Healthy Paths was originally designed and written by a multidisciplinary team comprised of psychologists and clinicians in close collaboration with individuals who were experiencing stressful life circumstances. Healthy Mind was aimed at managing stress and was not intended as an intervention for psychological disorders (e.g. depression, anxiety).

A game-based element was introduced to encourage continued usage of the app long enough for the intelligent triggering system to train (approximately two notification deliveries). Four starter tools were provided when first downloaded (see S2 Table). To unlock the five further tools, users were asked to rate the helpfulness of a tool each time it was viewed. Once unlocked, tools were accessible on demand; no restrictions were placed on when or how often users were able to access each tool.

All notifications consisted of a short teaser invitation (approximately 40 characters) to use one of the tools, which if clicked on, led to a further screen that offered more information about the suggested tool (see Table 1). This information screen was designed to support participants to overcome barriers to using the tools or provide interesting new facts about how and why the tool may be helpful. A range of different messages were developed for each tool in the app to ensure variability of content. The tool suggested within each notification was tailored to participants’ prior app usage and tool ratings. Three categories of notification were used: tool announcements, tool suggestions, and general reminders. Tool announcements invited participants to try out a newly unlocked tool. Tool suggestions encouraged re-use of tools that participants had previously found helpful. General reminders invited the participant to re-use the app rather than a specific tool. To minimise perceived repetitiveness, the tool suggested was varied between two consecutive notifications.

**Table 1:** Example notification messages.

Notification type	Teaser invitation	Information screen
Tool announcement	A new tool has been unlocked!	Congratulations, a new tool has been unlocked for you! It’s been a while since you’ve unlocked a new tool–that’s why we thought you might like a new one to try. Your new tool is: Body Scan. You can unlock all the Healthy Mind Tools just by using different tools and telling us what you think of them–each time you rate a tool we’ll unlock a new one for you. There are 9 tools to unlock.
Tool suggestion	Do you have 3 minutes?	When our lives are hectic we often forget to take some time to ourselves to relax and slow down. The great thing about taking a breathing space is that you can do it almost anywhere and all you need is 3 spare minutes! Click ‘next’ to give it a try.
General reminder	Take time to look after yourself today	We know it’s difficult to make time to use the Healthy Mind tools when there’s a lot going on in your life. But this is exactly the time when you need to look after yourself by doing things that help you to feel happier and healthier. It’s also why we’ve tried to make the Healthy Mind tools quick and easy to use–so that you don’t need to feel guilty for taking some time out. Click ‘next’ to give the Healthy Mind tools a try.

### Measures

At baseline, participants were asked to provide a valid email and complete a short demographic questionnaire including age, gender, and educational attainment. Baseline measures were kept intentionally short in order to mimic how individuals usually engage with apps. Usage of Healthy Mind was automatically recorded using the Life Guide Toolbox software. Table 2 provides a detailed description of the variables used to characterise notification response and intervention usage in the presented analysis. Semi-structured telephone interviews explored a) perceptions of Healthy Mind (e.g. likes, dislikes, reactions to notifications), and b) experiences of using Healthy Mind (e.g. specific tools used, time spent on the app, contexts of use) (see S2 Table for interview schedule). Field notes were taken during and after each interview to take note of any technical/usability issues with the Healthy Mind app as well as to capture early thoughts on potential codes for analysis.

**Table 2:** Variables characterising notification response and intervention usage.

Variable	Description
Notifications received	The number of notifications received.
Notifications viewed	The number of notifications viewed (*n*) and the percentage of notifications viewed relative to the number received (%).
Notifications actioned	The number of notifications (*n*) and the percentage of notifications (%) that were followed by the action suggested within the notification.
Response delay	The delay (in minutes) between when the notification was sent by the triggering system and when the notification was viewed by the user.
Logins (*n*)	The number of times participants opened the Healthy Mind app either spontaneously or via a notification.
Login duration	The length of time (in minutes) that participants spent on the app during each separate login.
Total duration	The length of time (in minutes) that participants spent on the app.
Tool completion	The number of times participants completed a Healthy Mind tool. Tools were defined as completed if participants viewed the ‘tool rating’ screen.
Days used	The number of days on which participants opened the Healthy Mind tool.
Ceased use	The proportion of participants who ceased use of Healthy Mind within 2 weeks after initial download.

### Analysis

Statistical analysis was performed using IBM SPSS Statistics for Windows 21 [34] on usage data collected within the first 2 weeks after initial app download. Means and standard deviations were computed for continuous variables and n/% computed for categorical variables. Since the sample size in this exploratory study did not provide sufficient power to definitively test for between group differences, results are interpreted as effect sizes with 95% confidence intervals [35,36]. Eta-square and Cohen’s *d* were computed as indications of effect size for continuous variables. Cramer’s *V* was computed as an indication of effect size for categorical variables.

All telephone interviews were audio-recorded and transcribed verbatim. Inductive thematic analysis was used to identify recurring patterns and themes relevant to understanding participants’ experiences of receiving notifications [37]. Data collection and analysis proceeded iteratively. The analysis was conducted by LM through a series of phases. First, transcripts were read and re-read then hand coded line-by-line using ‘in-vivo’ codes wherever possible. This preliminary set of codes were then organised into a set of potential themes. Constant comparison and deviant case analysis were used to identify data that did not fit within potential theme structure. Themes were subsequently added, merged and/or refined as appropriate. The final coding and theme structure was discussed and agreed with AW. A paper trail was maintained throughout all phases of analysis documenting progression from the raw data to the final theme structure and reported findings.

The analysis was conducted from a realist perspective, assuming that participants’ reports were a reflection of their genuine attitudes or experiences. This was an exploratory study and as such the primary analyst (LM) did not hold any pre-conceptions about what themes may emerge from the qualitative data. That said, the qualitative and quantitative analyses were conducted in parallel. It is therefore possible that emerging findings from the quantitative analyses influenced interpretation of the qualitative data and the relative salience of emerging themes.

## Results

### Sample characteristics

In total, 202 participants downloaded Healthy Mind and 162 were randomised to one of the three notification groups. 40 participants did not complete the baseline measures and so were not randomised to one of the three notification groups. An early technical error affected the first 85 randomised participants. 77 participants therefore provided usable data for the presented analysis (intelligent: *n* = 25; daily: *n* = 19; occasional: *n* = 33).

Just over half the participants were female (*n* = 48, 62%) with one participant declining to answer. Age data was missing or suspected to be false (i.e. default selected) for 8 participants. The age range of the remaining participants was 18 to 62 years (*M* = 35.94, *SD* = 10.54). Around half (*n* = 41, 53%) of the participants reported university level education (undergraduate or postgraduate degree), 6 (8%) reported A-level education, 13 (17%) reported GCSE level education, 8 (10%) reported attaining a diploma, vocational or professional qualification, 8 (10%) reported no formal educational qualifications, and 1 declined to answer.

### Notification response and intervention usage

On average, seven notifications were received (*M* = 7.03, *SD* = 4.94) and two notifications were viewed (*M* = 2.16, *SD* = 3.28) and subsequently actioned (*M* = 1.71, *SD* = 3.18). The average delay between receiving and viewing a notification was just under 3 hours (*M* = 163 minutes, *SD* = 362 minutes). Participants logged in to Healthy Mind between 1 and 26 times (*M* = 4.56, *SD* = 4.8) and used it on between 1 and 12 days (*M* = 2.96, *SD* = 2.55). Participants completed between 0 and 24 tools (*M* = 3.92, *SD* = 5.58). The average duration (*M*) of each login was 4 minutes (SD = 9 minutes) and average total duration of use 19 minutes (SD = 48 minutes). Just over half the participants stopped using Healthy Mind within 2 weeks post-download (*n* = 36, 53%). Table 3 presents descriptive statistics (*M*, *SD*) for notification response and app usage by group.

**Table 3:** Descriptive statistics for notification response and app usage by group, M (SD).

	Intelligent	Daily	Occasional
Notifications received	8.08 (6.17)	10.00 (5.28)	4.52 (1.03)
Notifications actioned (*n*)	2.60 (4.41)	2.05 (3.41)	.85 (1.20)
Notifications viewed (*n*)	2.92 (4.47)	2.63 (3.69)	1.3 (1.24)
Logins	5.44 (7.03)	4.89 (4.14)	3.7 (2.57)
Days used	3.04 (2.94)	3.63 (3.30)	2.52 (1.52)
Response delay (min)	252 (532)	195 (327)	67 (108)
Login duration (mins)	4 (3)	3 (3)	6 (13)
Total duration (mins)	19 (52)	13 (19)	23 (55)
Notifications actioned (%)	25.17 (28.52)	19.00 (26.37)	18.94 (27.83)
Notifications viewed (%)	29.80 (29.06)	28.05 (32.49)	30.45 (30.42)
Tools completion	4.04 (7.05)	3.47 (3.94)	4.09 (5.28)

Table 4 presents Cohen’s *d* for pairwise group comparisons where η^2^ ≥.01 (small effect). Medium effects of group were found for the number of notifications viewed, η^2^ = .14 (95% CI .00 - .33), and actioned, η^2^ = .15 (95% CI .00 - .35). The intelligent and daily groups appeared to view more notifications (medium effect), and take action on more notifications (medium effect) compared with the occasional group. Medium effects of group were also found for the number of logins, η^2^ = .06 (95% CI .00 - .22), the number of days on which the app was used, η^2^ = .06 (95% CI .00 - .22), and delay in viewing a notification, η^2^ = .05 (95% CI .00 - .18). The intelligent and daily groups appeared to log into the app more (small effect) and have a shorter response delay (small-medium effect) than the occasional group. The daily group also appeared to use the app on a greater number of days than the occasional group (small-medium effect).

**Table 4:** Effect sizes for group comparisons on notification response and app usage.

	Intelligent vs daily		Intelligent vs occasional		Daily vs occasional	
	d	95% CI	d	95% CI	d	95% CI
Notifications received	-.33*	-.93, .27	.76***	.19, 1.31	1.29***	.58, 1.98
Notifications actioned (*n*)	.14	-.46, .73	.50**	-.04, 1.04	.43**	-.16, 1.00
Notifications viewed (*n*)	.07	-.53, .67	.47**	-.07, 1.00	.44**	-.15, 1.01
Logins	.09	-.51, .69	.31*	-.22, .84	.33*	-.25, .90
Days used	.19	-.79, .41	.24*	-.29, .75	.40**	-.18, .97
Response delay (min)	.10	-.49, .70	.38**	-.15, .91	.39**	-.20, .97
Login duration (mins)	.29*	-.31, .89	-.21*	-.72, .32	-.28*	-.85, .29
Total duration (mins)	.16	-.44, .75	-.07	-.59, .45	-.23*	-.79, .34
Notifications actioned (%)	.23*	-.37, .82	.22*	-.30, .74	.00	-.12, .12

Note.

*** denotes large effect (≥ .8)

** denotes medium (≥ .5) and small-medium effect (≥ .35) and

* denotes small effect (≥ .2) according to Cohen’s guidelines [38].

A small effect of group was found for duration of each login, η^2^ = .02 (95% CI .00 - .10), total duration of app usage, η^2^ = .01 (95% CI .00 - .06), and the percentage of notifications actioned, η^2^ = .01 (95% CI .00 - .08). Duration of app use appeared to be shorter in the daily group compared with the occasional group (small effects). The intelligent group appeared to take action on a greater percentage of notifications compared with both the daily and occasional groups (small effect). A small effect of group was also found for the proportion of participants ceasing use of Healthy Mind within 2 weeks after initial download, Cramer’s *V* = .19. A higher proportion of participants in the intelligent group appeared to cease use of Healthy Mind (*n* = 15, 60%) compared with the daily (*n* = 8, 42%, Cramer’s *V* = .18) and occasional (*n* = 13, 39%, Cramer’s *V* = .20) groups. Similar proportions of participants appeared to cease use of Healthy Mind in the daily and occasional groups (Cramer’s *V* = .03).

The effect of group on the percentage of notifications viewed, η^2^ = .00 (95% CI .00 - .02), and tool completion was negligible, η^2^ = .00 (95% CI .00 - .04).

### Intervention experiences

Seven participants provided consent to be interviewed; 6 participants were subsequently interviewed (intelligent: *n* = 2, occasional: *n* = 4) with 1 providing no response to contacts from the research team. All 6 participants were female, aged between 21 to 52 years of age (*M* = 34.17, *SD* = 11.44). Most were educated to at least degree level (*n* = 5, 83%). Two participants were affected by the early technical error affecting intended delivery of notifications. However, they were included in the qualitative analysis as their experiences of using Healthy Mind could nevertheless provide useful insights of app engagement. Three themes provide insight into participants’ experiences of notifications.

#### Notification awareness

A small number of participants accurately reported on the timing and frequency of notification delivery. Other participants appeared to be unaware of the notification delivery schedule or reported inaccurate perceptions. Some participants commented that they were happy with the number and type of notifications received. Others described experiencing frustration in response to a perceived lack of variety in the notification content:

“And then in the end it got me a bit annoyed, ‘cause I was like, ‘Oh, I’ve done this already—come on, you know, if you’re going to send me a reminder, like, it’ll be nice if it was something different.” (P12)

Notifications appeared to be one of a range of factors that encouraged participants to use the app. Most participants appeared to perceive the notifications as a reminder to use the Healthy Mind tools. Participants commented that notifications encouraged them to take time out or stop and think about their day.

“So it’s been really useful for, ‘cause I’m really busy, have two jobs and children and lots of other stuff, so sometimes you just forget to take time for yourself so…, and getting that reminder, as well, is really good. That kind of thing, oh yeah, I should have a few minutes just to sort myself out.” (P08)

Participants differed in the extent to which they reported relying on the notifications; some participants reported using the app only in response to notifications (but not necessarily after every notification), others reported spontaneous use of the app. One participant explained that whilst she did not rely on the notifications to remind her to use the tools, the notifications did prompt her to consider how helpful the tools had been.

#### Changing relationships

A few participants discussed how the usefulness of notifications lessened as they became familiar with the app content and more experienced with using the Healthy Mind tools. One participant explained that after an initial learning period, the tools were used as and when needed rather than in response to a notification and often without the need to access Healthy Mind. Participants also described quickly working out and sticking to their favoured tools.

“When I first started using the app, I was using the app and kind of like responding to the prompts, and then, as I’ve kind of practiced a bit more, I don’t tend to, like, use the actual app as much–it’s just more that I’ve kind of learnt the techniques that it’s taught me, and I use them as I need. So yeah, I’d kind of say like my relationship kind of, like, changed.” (P09)

#### Context and fit

The contexts in which notifications were responded to varied between each participant. Some participants reported using the app and picking up notifications in the evening to reflect on the day. Other participants reported using the app as a positive start to the day, during the working day while travelling, or only in response to stressful experiences. Another participant described using the app while commuting on public transport, which constrained use of some of the tools.

“But you know what, I’ve not, I’ve never tried to do that [Healthy Mind] in the right conditions, I think, I’ve kind of thought ‘oh, I’ve got twenty five minutes on the [train], perhaps we’ll do it then,’ but I kind of feel self-conscious …. So again, what, how I haven’t probably used it is in the privacy of my own home, sitting down really to kind of go through it, understand about that sort of, learn that kind of relaxation technique, and, and use it in, use it in that way.” (P11)

Most of the participants reported picking up notifications at times that they perceived to be most useful or convenient, not necessarily when the app sent them through. Indeed, a couple of participants discussed their appreciation of the tone of the notifications, which they perceived to offer suggestions rather than overt demands for immediate action.

“It wasn’t kind of like, ‘oh, you’ve got to do this now’ you know? It wasn’t kind of making demands on your time, it was just kind of like reminding you that, like, these are things that help you to kind of fight stress.” (P09)

## Discussion

In terms of timing, no meaningful differences were found between intelligent, sensor driven and pre-determined, static notification delivery. This counters conclusions drawn from prior research where sensor-driven models have shown slight advantages over non-sensor-driven comparators [24]. However, prior research has examined sensor-driven models in artificial experimental settings where participants were incentivised to respond accurately to arbitrary, survey-based notifications. The contrasting pattern of results observed in the current study highlights the need to evaluate emerging sensor-driven intervention models in a variety of contexts, particularly real-world use. In terms of frequency, more notifications were viewed and actioned in the intelligent and daily groups compared to the occasional group. The percentage of notifications viewed and actioned appeared equivalent across groups, as did the number of Healthy Mind tools completed. This suggests that sending frequent, daily notifications may not have adverse effects on response rate, nor does it seem to deter app usage. Sending frequent, daily notifications also means that users are likely to see more intervention content.

Participants in this study appeared to pay little conscious attention to the frequency and timing of notifications–instead some were demotivated by the perceived repetitiveness of the notification content despite attempts to provide variety. The influence of notification content has been noted previously [20] and highlights the need to adequately pilot content [39] to ensure that it provides a sufficiently interesting and rewarding experience [40].

Response to notifications and usage of Healthy Mind was low across the three notification groups. On average, participants opened notifications a few hours after receipt and stopped using Healthy Mind after a few days. It has been suggested by previous research that perceived social pressure may drive notification response [25]. Indeed, qualitative data from the current study indicated that notifications were perceived as suggestions for actions that can be ignored or deferred to a later time, as needed. More frequent response to notifications may be seen for interventions that incorporate an explicit social or support-based component. Participants in the current study also described ceasing use of Healthy Mind once they were familiar with the tools available. This pattern of usage fits with prior qualitative research highlighting individuals’ tendency to use apps fleetingly [16] or to “outgrow” apps [41]. A low-intensity, short-term pattern of usage is not necessarily problematic for all app-based behaviour change interventions. A few days of quick logins may be sufficient to enable users to learn new tools that can then be practiced without guidance from an intervention.

### Limitations

The sample size did not offer sufficient power to definitively test for between group differences. The effect sizes reported in this study should be considered tentative and no conclusions were drawn from small effects given that all confidence intervals crossed zero. The qualitative sample was also not sufficient to achieve saturation or to compare experiences across the different notification groups. Explanations for the data in this study are hypothesis generating only and should be used to stimulate further empirical research. While the accuracy of the intelligent triggering system has been tested and reported elsewhere [24] it was not explicitly tested within this study. Previous tests of the intelligent triggering system also examined user interruptibility independently from intervention content. Different notification types (e.g. tool announcements vs. tool suggestions) may be associated with varied response rates. Notification content could not be examined experimentally in this study since the frequency of each notification type varied according to app usage patterns. New libraries for content-driven notifications have recently been developed [42]. Further empirical research is needed to examine the effect of notification content and purpose on user receptivity and response in the context of health behaviour interventions.

The design of this study did not permit us to examine the effect of notification group on perceived stress or other health-related outcomes. Further research in a larger sample over a more extended period is needed to identify whether frequency or timing of notification delivery is associated with health-related change. Finally, this study examined the impact of notification timing and frequency for one specific intervention, with one specific implementation of intelligent sensor-driven notifications. Additional research is needed to examine whether the same pattern of results is observed for other interventions that may have varied aims, target behaviour(s) and populations, content, notification types, and sensor-driven data models.

### Implications

The results from this study suggest that, in naturalistic settings, tailoring notification delivery to location, movement, and time of day may not always offer any advantage over a priori assumptions about convenient moments. Smartphones offer a wide range of contextual data that were not utilised in the current study. It may be that alternative combinations of sensor data will enhance response rates and intervention usage. The results from this study also suggest that sending frequent, daily notifications may not deter users from engaging with an app-based intervention and could mean that they are exposed to more of the intervention content. However, precise thresholds for the frequency at which notifications deter or encourage intervention usage are not yet known. For example, it may be that while daily notifications are acceptable, several notifications per day may be unacceptable. Similarly, too many intelligent, sensor-driven notifications may be perceived by users as random. Optimal thresholds may also vary for different population sub-groups and health behaviours. Larger scale studies are needed to test the hypotheses generated from this study and to examine the impact of other combinations of sensor data and different notification delivery schedules.

Current approaches to measuring intervention engagement typically rely on objectively recorded usage data, which may underestimate engagement with the intervention content. It may be that initial notification receipt or observed app usage provided reminders to practice relevant tools at a later time. Subsequent practice of the tool will not be reflected in the observed usage patterns. Identifying variables that indicate optimal receptivity to intervention content is an ongoing challenge for the development of just-in-time adaptive interventions [43]. Nested qualitative studies can provide more in-depth insight of participants’ experiences following the intervention and their potential reasons for continued engagement or disengagement [33]. Adoption of a mixed-methods approach to evaluating digital interventions can support more informed and appropriate conceptualisations about what constitutes poor versus successful engagement and the factors that underlie whether and when an individual stops using an intervention. Additional work is needed to identify and evaluate novel methods for assessing engagement with digital interventions that can capture off-line activities and experiences.

### Conclusion

This exploratory study suggests that tailoring the delivery of notifications based on users’ current location and movement may not always encourage greater response rates or intervention usage in a naturalistic setting compared to sending notifications at assumed good times. This study also suggests that sending frequent, daily notifications may enhance exposure to intervention content without deterring continued engagement. Additional research is needed to test the hypotheses generated from this study and to examine whether other types and combinations of phone-based sensor data can enhance the delivery of notifications and subsequent behaviour change within different health behaviour change interventions. Mixed methods approaches that combine quantitative and qualitative data can provide a clearer and more comprehensive picture of user engagement with health behaviour change interventions.

## Supporting Information

S1 Table Healthy Mind Tools. (DOCX)

S2 Table Interview Schedule. (DOCX)
