# Correction: The Effect of Timing and Frequency of Push Notifications on Usage of a Smartphone-Based Stress Management Intervention: An Exploratory Trial

**DOI:** 10.1371/journal.pone.0198008

**Published:** 2018-05-22

**Authors:** Leanne G. Morrison, Charlie Hargood, Veljko Pejovic, Adam W. A. Geraghty, Scott Lloyd, Natalie Goodman, Danius T. Michaelides, Anna Weston, Mirco Musolesi, Mark J. Weal, Lucy Yardley

The affiliation for the eighth author is incorrect. Anna Weston is not affiliated with #3 but with #10 Electronics and Computer Science, University of Southampton, Southampton, Hampshire, UK.
