# Supplementary material for: The Effect of Timing and Frequency of Push Notifications on Usage of a Smartphone-Based Stress Management Intervention: An Exploratory Trial
Source: PLoS One. 2017 Jan 3;12(1):e0169162. doi: 10.1371/journal.pone.0169162 (PMC5207732; doi:10.1371/journal.pone.0169162)
Supplement: S1 Table — (DOCX) [file pone.0169162.s001.docx]

**S1 Table. Healthy Mind Tools.**

| Tool | Description |
| --- | --- |
| *Walking with Awareness | Guided walking activity to encourage greater conscious awareness of the experience of walking (e.g. bodily sensations, surrounding environment). |
| My Daily Reactions | Identify and record physical, behavioural, and affective reactions to daily stressful events. |
| *3 Minute Breathing Space | Guided 3-minute breathing exercise. |
| Body Scan | Guided 10-minute body scan exercise. |
| *Connect with Others | Create/select from plans to spend time with other people. |
| Enjoyable Moments | Record and reflect on how often one engages in pleasant activities (e.g. reading, taking a long bath, gardening). |
| Sleep Well | Select and review goals for improving sleep quality. |
| *Self-Kindness | Guided exercises to cultivate self-compassion. |
| Positive Thinking | Create/select from a list of positive thoughts (e.g. I always learn something new from dealing with a stressful situation). |

* Starter tool.
