# Supplementary material for: The Effect of Timing and Frequency of Push Notifications on Usage of a Smartphone-Based Stress Management Intervention: An Exploratory Trial
Source: PLoS One. 2017 Jan 3;12(1):e0169162. doi: 10.1371/journal.pone.0169162 (PMC5207732; doi:10.1371/journal.pone.0169162)
Supplement: S2 Table — (DOCX) [file pone.0169162.s002.docx]

**S2 Table. Interview Schedule.**

| Introduction |
| --- |
| - Introduce self by name/affiliation and explain that you are a researcher working on the Healthy Mind project. Thank participant for their time. - Check participants comfort and explain that calls normally last between 20 – 60 minutes. Remind the participants that the call will be recorded. - Explain that hearing about their experiences will help us to understand how helpful the Healthy Mind tool is and how we might improve it in the future. - Reassure the participant that there are no right or wrong answers and they can feel free to tell you about any thoughts or comments they have, even if they seem unimportant. - Explain to the participant that you are interested in every aspect of their experience of Healthy Mind, good or bad. |
| Opening questions and prompts |
| How have you been getting on with Healthy Mind?  Can you tell me what it’s been like using Healthy Mind?   - *Can you describe any of the things you liked about it?* - *Can you describe any of the things you didn’t like about it?* - *Can you tell me more about what you thought of the different tools in the app?* - *Can you tell me about any problems you’ve experienced?*   Can you tell me a bit about how much you’ve used Healthy Mind?   - *How much time have you spent using the app?* - *Can you describe when you tended to use the app?*   Can you talk me through what you have been doing/what have you done on the app?  How did you feel about getting notifications from Healthy Mind?   - *How often did you get notifications?* - *How did you react when you got a notification?* - *What did you think of the notifications?*   Can you tell me about how you came to hear about Healthy Mind?  Overall, what did you think of Healthy Mind? |
